# Supplementary material for: Co-Infection of Tobacco Rattle and Cycas Necrotic Stunt Viruses in Paeonia lactiflora: Detection Strategies, Potential Origins of Infection, and Implications for Paeonia Germplasm Conservation
Source: Viruses. 2024 May 31;16(6):893. doi: 10.3390/v16060893 (PMC11209033; doi:10.3390/v16060893)
Supplement: Supplementary file 1 [file viruses-16-00893-s001.zip › viruses-3015339-supplementary.pdf]

Supplementary material:
These are Electronic Supplementary Materials for the article: **Co-infection of tobacco rattle and cycas necrotic stunt viruses in *Paeonia lactiflora*: detection strategies, potential origins of infection, and implications for *Paeonia* germplasm conservation** by Nastassia B. Vlasava (vlasava@umich.edu), David C. Michener, Siarhei Kharytonchyk, Liliana Cortés-Ortiz, accepted for publication in the special issue of *Viruses* "Rapid and accurate detection of plant pathogens towards improving biovigilance-based crop management strategies" (30th of April, 2024)**.**

A

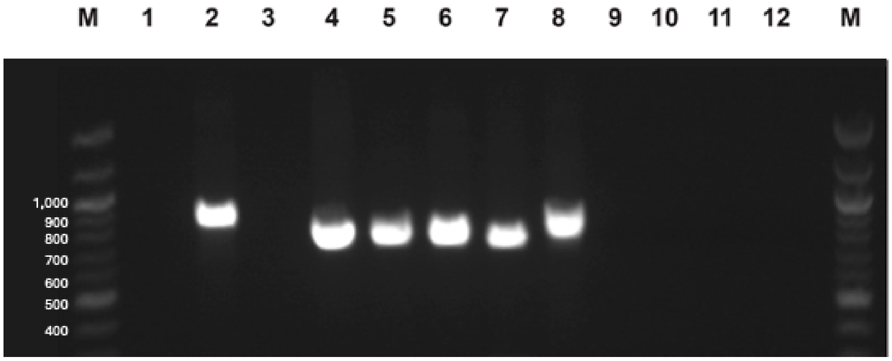

B

**Name of cultivar (and abbreviation[a])**	**Years of observation**		
Duchess of Portland (DP-1)	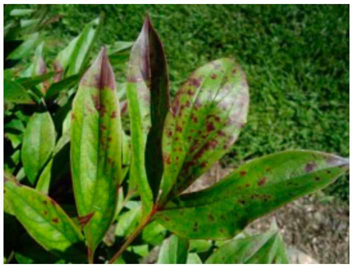 DP1-2015 *	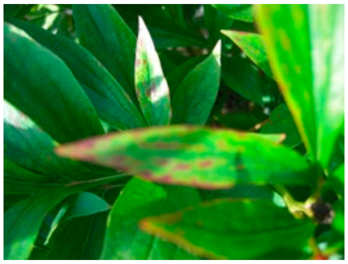 DP1-2016	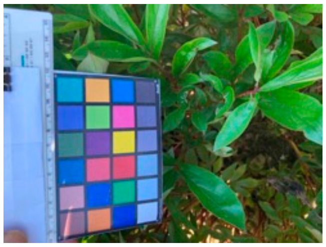 DP1-2024
Duchess of Portland (DP-2)	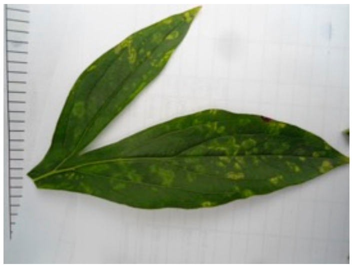 DP2-2015 *	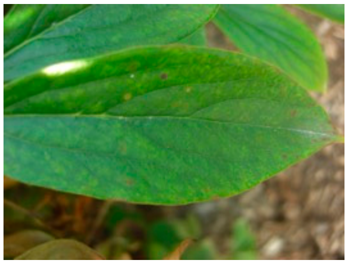 DP2-2016	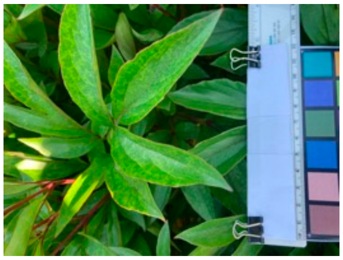 DP2-2024

Gisele (Gis1)

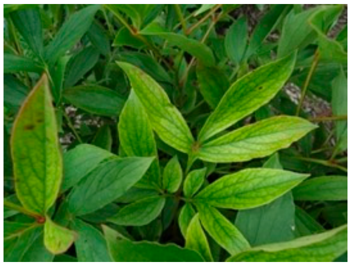

Gis-1-2015 *

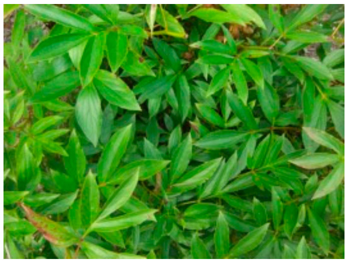

Gis1-2016 *

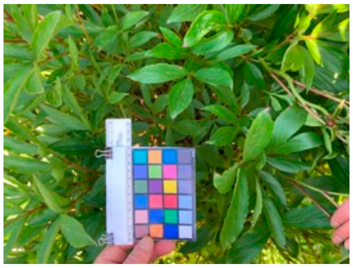

Gis1-2024

Gisele (Gis2)

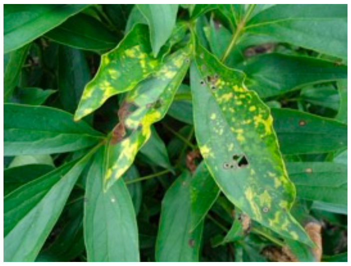

Gis2-2015 *

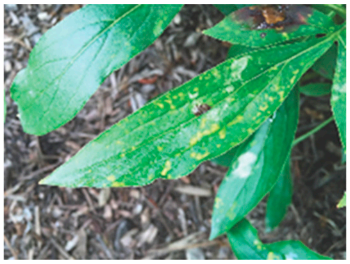

Gis2-2016 *

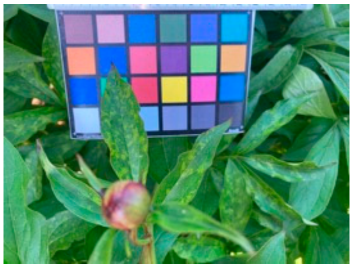

Gis2-2024

Yeso (Y1)

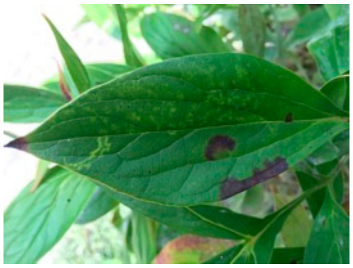

Y1-2016 *

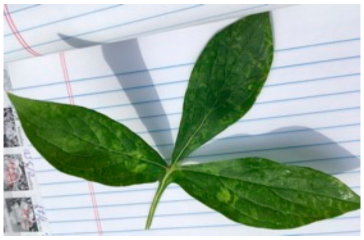

Y1-2017

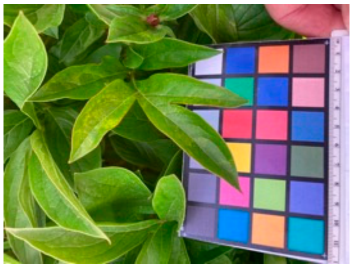

Y1-2024

Yeso (Y2)

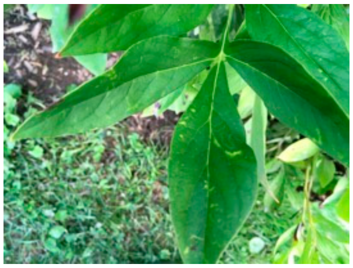

Y2-2016 *

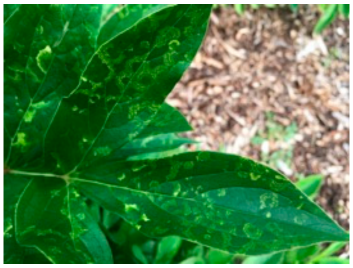

Y2-2017

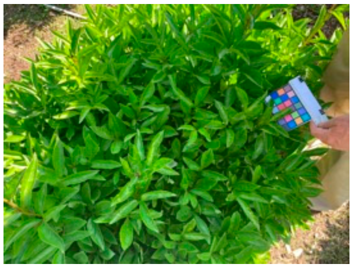

Y2-2024

Gigantea (G1)

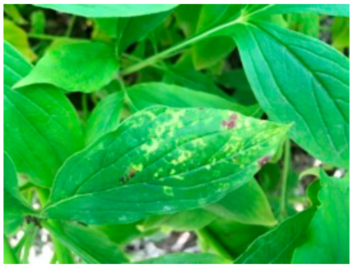

G1-2016 *

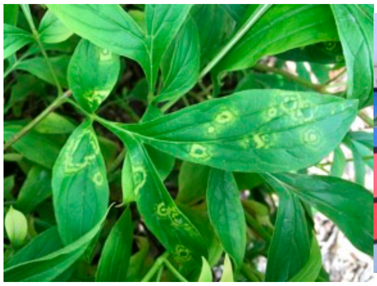

G1-2018

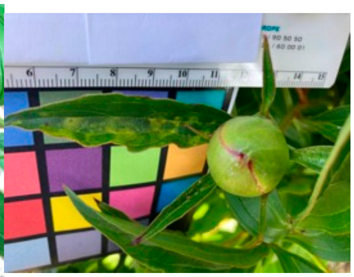

G1-2024

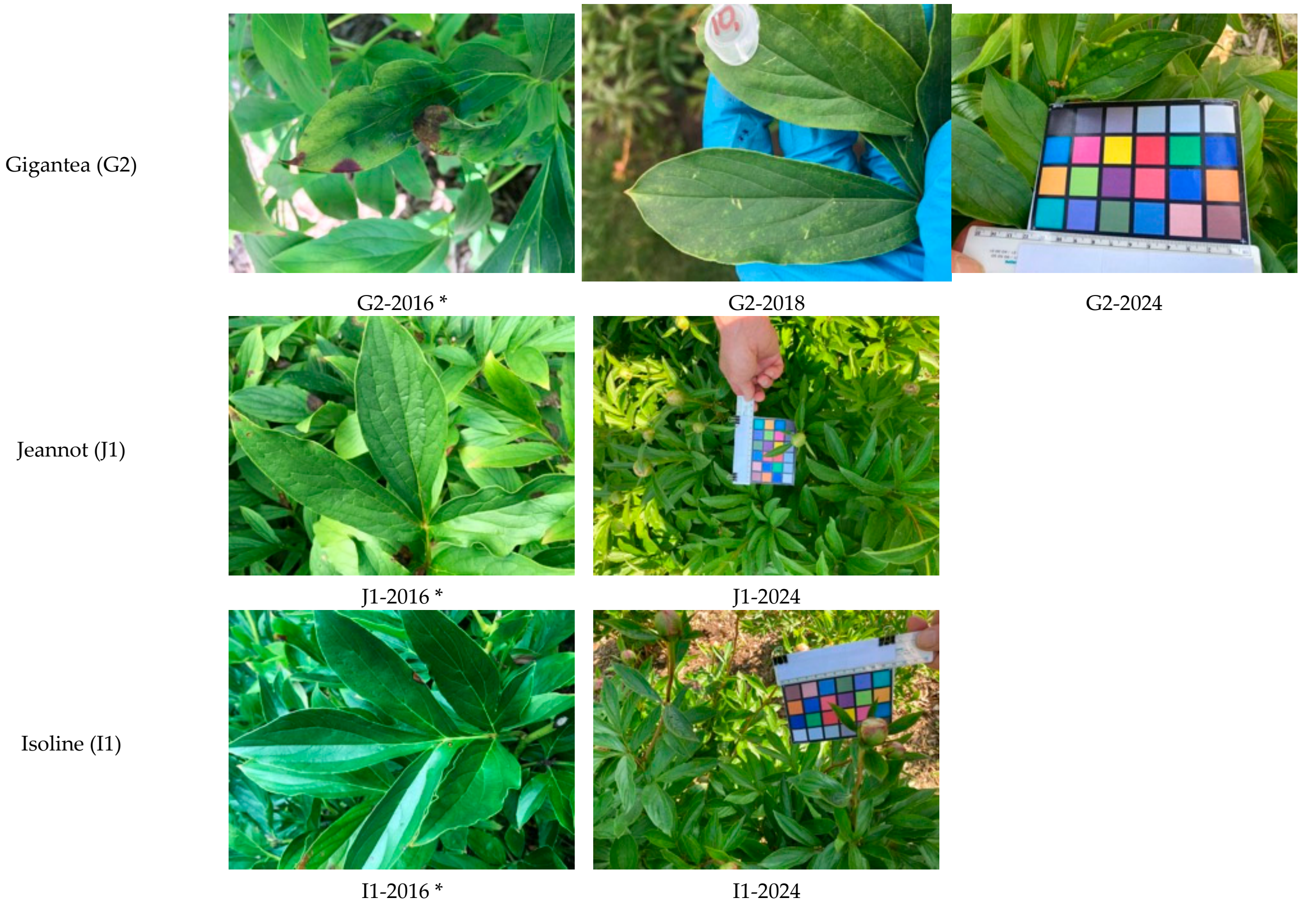

Figure S1. TRV detection in *P. lactiflora* plants sampled at the University of Michigan Nichols Arboretum's peony living collection**.** **(A)** Representative PCR detection of TRV from cDNAs synthesized from total RNA with TRV specific primers (Table 1). 1 = DP1-2015, 2 = DP2-2015, 3 = Gis1-2016, 4 = Gis2-2016, 5 = Y1-2016, 6 = Y2-2016, 7 = G1-2016, 8 = G2-2016, 9 = J1-2016, 10 = I1-2016 (see Table 2 for abbreviations). Symptomatic plants exhibiting leaf mottle mosaic symptoms (2, 4–8), asymptomatic plants (1, 3, 9, 10). Lanes 11 and 12 are negative PCR and negative RT controls, respectively. M = 100 bp DNA size standard ladder (New England Biolab). Electrophoresis was performed in 1% agarose at 100 volts for 60 min in 1X TAE buffer. Visualizing of PCR products were performed by adding 6×GelRed Prestain Loading Buffer (Biotium, USA) to the sample before applying to gel. TRV-specific amplicons are ~779 bp. **(B)** Series of photographs of leaf symptoms on the plants in this study in the year when samples were collected and up to now. Asterix (*) indicates plants and the year of sample collection used for RNA extractions of TRV (Figure S1A) and CNSV (Figure S2) identification in this study.

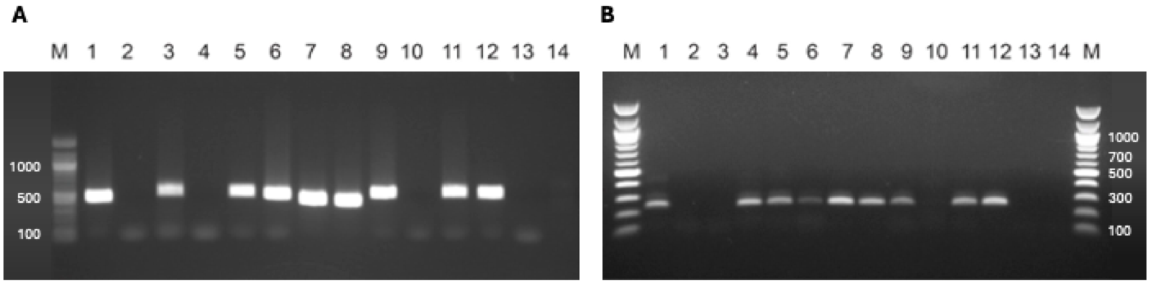

Figure S2. Representative RT-PCR detection of CNSV from cDNAs synthesized from total RNA from *P. lactiflora* plants, sampled at MBGNA peony field collection (lanes 1–12). Negative (water) PCR control and negative RT control are shown in lanes 13 and 14, respectively. M = 100 bp DNA ladder (New England Biolab). **(A)** PCR amplification of polyprotein 1 gene fragment (~380 bp) with new NepoPl primers (Table 1); 1 = DP1-2015, 2 = DP2-2015, 3 = Gis1-2015, 4 = Gis2-2015, 5 = Gis1-2016, 6 = Gis2-2016, 7 = Y1-2016, 8 = Y2-2016, 9 = G1-2016, 10 = G2-2016, 11 = J1-2016, 12 = I1-2016 (see Table 2 for abbreviations). Visualizing of PCR products were performed by adding 6×GelRed Prestain Loading Buffer (Biotium, USA) to the sample before applying to gel. **(B)** PCR amplification of RdRp gene (~250 bp) with Nepo family Type B (Table 1). 1 = DP1-2015, 2 = DP2-2015, 3 = Gis2-2015, 4 = Gis1-2015, 5 = Gis1-2016, 6 = Gis2-2016, 7 = Y1-2016, 8 = Y2-2016, 9 = G1-2016, 10 = G2-2016, 11 = J1-2016, 12 = I1-2016 (see Table 2 for abbreviations). Staining of PCR products were performed using 0.25 µg/mL of Ethidium Bromide (Alfa Aestar).

Electrophoresis was performed in 1% agarose at 100 volts for 60 min in 1X TAE buffer. CNSV-specific amplicons are ~380 **(A)** and 250 bp **(B)**.

Table S1. Sequences of TRV isolates and Pea Early Browning Virus (PEBV) used for phylogenetic analysis (ordered as they appear on the tree in Fig. 2). USA = United States of America, UK = United Kingdom, OH = Ohio, AK = Alaska, OR = Oregon, MI = Michigan. Sequences generated in this study are in bold font.

**Isolate name**	**Country, state**	**Host species**	**Year of GenBank submission or sample collection ([a])**	**GenBank accession number**
11r21	Poland	*Solanum tuberosum*	2010	KF758790
1AKBH	USA, AK	*Dicentra spectabilis*	2010	JX912715
20NR002	Canada	*Epimedium*	2020[a]	MZ344591
AL	Germany	*Alstroemeria aurea*	2011	HM195288
ALB6-Tobra1	USA, OH	*Hosta sieboldii cv. Albo-marginata*	2009[a]	JX267265
ALB6-Tobra2	USA, OH	*Hosta sieboldii cv. Albo-marginata*	2009[a]	JX267266
ALB6-Tobra3	USA, OH	*Hosta sieboldii cv. Albo-marginata*	2009[a]	JX267267
ALB6-Tobra4	USA, OH	*Hosta sieboldii cv. Albo-marginata*	2009[a]	JX267268
ALB6-TRV1	USA, OH	*Hosta sieboldii cv. Albo-marginata*	2009[a]	JX267269
ALB6-TRV2	USA, OH	*Hosta sieboldii cv. Albo-marginata*	2009a	JX267270
ALB6-TRV3	USA, OH	*Hosta sieboldii cv. Albo-marginata*	2009[a]	JX267271
ALB6-TRV4	USA, OH	*Hosta sieboldii cv. Albo-marginata*	2009[a]	JX267264
B8	China	*Lilium oriental*	2018	MH360253
ByKt(LS)	Germany	*Solanum tuberosum*	2015	KT033405
ByKt(Bav)	Germany	*Solanum tuberosum*	2015	KT033407
Deb57	Poland	*Solanum tuberosum*	2009	KF758791
DicentraAsympt1-21Tobra	USA, OH	*Dicentra spectabilis*	2010[a]	JX627774
DicentraAsympt1-23Tobra	USA, OH	*Dicentra spectabilis*	2010[a]	JX627775
DicentraAsympt1-27Tobra	USA, OH	*Dicentra spectabilis*	2010[a]	JX627776
DicentraAsympt1-29Tobra	USA, OH	*Dicentra spectabilis*	2010[a]	JX627777
DicentraAsympt1-30Tobra	USA, OH	*Dicentra spectabilis*	2010[a]	JX627778
DicentraAsympt1-32TRV	USA, OH	*Dicentra spectabilis*	2010[a]	JX627779
DicentraAsympt1-35TRV	USA, OH	*Dicentra spectabilis*	2010[a]	JX627780
DicentraAsympt1-37TRV	USA, OH	*Dicentra spectabilis*	2010[a]	JX627781
DicentraAsympt1-39TRV	USA, OH	*Dicentra spectabilis*	2010[a]	JX627782
DicentraAsympt1-40TRV	USA, OH	*Dicentra spectabilis*	2010[a]	JX627783
DicentraRutgers2-11Tobra	USA, OH	*Dicentra spectabilis*	2010[a]	JX627784
DicentraRutgers2-	USA, OH	*Dicentra spectabilis*	2010[a]	JX627785

12Tobra				
DicentraRutgers2-13Tobra	USA, OH	*Dicentra spectabilis*	2010[a]	JX627786
DicentraRutgers2-15Tobra	USA, OH	*Dicentra spectabilis*	2010[a]	JX627787
DicentraRutgers2-17Tobra	USA, OH	*Dicentra spectabilis*	2010[a]	JX627788
DicentraRutgers2-21TRV	USA, OH	*Dicentra spectabilis*	2010[a]	JX627789
DicentraRutgers2-23TRV	USA, OH	*Dicentra spectabilis*	2010[a]	JX627790
DicentraRutgers2-27TRV	USA, OH	*Dicentra spectabilis*	2010[a]	JX627791
DicentraRutgers2-28TRV	USA, OH	*Dicentra spectabilis*	2010[a]	JX627792
DicentraSympt1-13TRV	USA, OH	*Dicentra spectabilis*	2010[a]	JX627798
DicentraSympt1-14TRV	USA, OH	*Dicentra spectabilis*	2010[a]	JX627799
DicentraSympt1-15TRV	USA, OH	*Dicentra spectabilis*	2010[a]	JX627800
DicentraSympt1-17TRV	USA, OH	*Dicentra spectabilis*	2010[a]	JX627801
DicentraSympt1-18TRV	USA, OH	*Dicentra spectabilis*	2010[a]	JX627802
DicentraSympt1-1Tobra	USA, OH	*Dicentra spectabilis*	2010[a]	JX627793
DicentraSympt1-4Tobra	USA, OH	*Dicentra spectabilis*	2010[a]	JX627794
DicentraSympt1-6Tobra	USA, OH	*Dicentra spectabilis*	2010[a]	JX627795
DicentraSympt1-7Tobra	USA, OH	*Dicentra spectabilis*	2010[a]	JX627796
DicentraSympt1-8Tobra	USA, OH	*Dicentra spectabilis*	2010[a]	JX627797
DicentraSympt2-1TRV	USA, OH	*Dicentra spectabilis*	2010[a]	JX627803
DicentraSympt2-4TRV	USA, OH	*Dicentra spectabilis*	2010[a]	JX627805
DicentraSympt2-6TRV	USA, OH	*Dicentra spectabilis*	2010[a]	JX627806
DicentraSympt2-7TRV	USA, OH	*Dicentra spectabilis*	2010[a]	JX627773
DSMZ PV-0043	Germany (?)	*Helianthus annuus (Lab host Nicotiana tabacum*	2021	MW854258
DSMZ PV-1227	Germany	*Narcissus*	2021	MW854287
DSMZ PV-1229	Netherlands	*Anemone*	2021	MW854289
HaB	Germany	*Nicotiana benthamiana*	2013	KJ826365
Ho	Germany	*Hosta sp.*	2006[a]	JQ235203
Chinese-31	China	*Paeonia lactiflora*	2016	KX370822
Chinese-28	China	*Paeonia lactiflora*	2016	KX370821
Ina	Germany	*Solanum tuberosum*	2015	KT033403
JT2	Finland	*Lysimachia nummularia*	2014	MK580817
JEO11-22	Burundi	*Solanum tuberosum cv. Ndinamagara*	2016[a]	MT537600
JEO11-24	Burundi	*Solanum tuberosum cv. Ndinamagara*	2016[a]	MT537601
MI-1	USA, MI	*Solanum tuberosum*	2007[a]	GQ903771
Mirow	Germany	*Solanum tuberosum*	~1960[a]	KT734612
MPA	Germany	*Solanum tuberosum*	2014[a]	KT020851
Mlo7	Poland	*Solanum tuberosum*	2010	KF758792
ORY	USA, OR	*Nicotiana clevelandii*	1998	AF034622
**Paela DP/p1-2015** (DP1-2015a*)	**USA, MI**	***Paeonia lactiflora***	**2015[a]**	**MF918561**
**Paela Gisele/p1-2015** (Gis1-2015*)	**USA, MI**	***Paeonia lactiflora***	**2015[a]**	**MF918562**
**Paela Gisele/p2-2016** (Gis2-2016*)	**USA, MI**	***Paeonia lactiflora***	**2016[a]**	**MF918563**
**Paela Yeso/p2-2016** (Y2-2016*)	**USA, MI**	***Paeonia lactiflora***	**2016[a]**	**MF918564**
**Paela Yeso/p1-2016** (Y1-	**USA, MI**	***Paeonia lactiflora***	**2016[a]**	**MF918565**

2016 *)				
**Paela Gigantea/p1-2016** (G1-2016*)	**USA, MI**	***Paeonia lactiflora***	**2016[a]**	**MF918566**
**Paela Gigantea/p2-2016** (G2-2015*)	**USA, MI**	***Paeonia lactiflora***	**2016[a]**	**MF918567**
PeonyTRV1	USA, OH	*Paeonia lactiflora*	2009[a]	JX144387
PeonyTRV2	USA, OH	*Paeonia lactiflora*	2009[a]	JX144388
PeonyTRV3	USA, OH	*Paeonia lactiflora*	2009[a]	JX144389
PeonyTRV4	USA, OH	*Paeonia lactiflora*	2009[a]	JX144390
PeonyTRV5	USA, OH	*Paeonia lactiflora*	2009[a]	JX144382
PeonyTobra1	USA, OH	*Paeonia lactiflora*	2009[a]	JX144383
PeonyTobra2	USA, OH	*Paeonia lactiflora*	2009[a]	JX144384
PeonyTobra3	USA, OH	*Paeonia lactiflora*	2009[a]	JX144385
PeonyTobra5	USA, OH	*Paeonia lactiflora*	2009[a]	JX144386
PVS-340-Tobra-35	USA, OH	*Epimedium sp.*	2013	KF705615
PVS-340-TRV-26	USA, OH	*Epimedium sp.*	2013	KF705616
PVS-620-Tobra-28	USA, OH	*Epimedium sp.*	2013	KF705617
PVS-620-TRV-20	USA, OH	*Epimedium sp.*	2013	KF705618
PpK20	Netherlands	Unknown[b]	2001	AF314165
PpK20_2	Netherlands	Unknown[b]	2004	AF166084
Ppk20_3	USA	Unknown[b]	2002	AF406990
PpO85 (Hel)	Netherlands	*Paratrichodorus pachydermus*[a]	2003	AJ586803
RHS	UK	*Allium caeruleum*	2015[a]	KT223102
Rostock	Germany	*Solanum tuberosum*	2015	KT734611
SHM	Germany	*Solanum tuberosum*	2015	KT033406
Slu24	Poland	*Solanum tuberosum*	2009	KF758793
SOS6-TRV10	USA, OH	*Hosta sp.*	2009[a]	JX267272
SOS6-Tobra5	USA, OH	*Hosta sp.*	2009[a]	JX267276
SOS6-TRV3	USA, OH	*Hosta sp.cv. So Sweet 6*	2009[a]	JX267277
SOS6-TRV5	USA, OH	*Hosta sp.*	2009[a]	JX267278
SOS6-TRV6	USA, OH	*Hosta sp.*	2009[a]	JX267279
SOS6-TRV7	USA, OH	*Hosta sp.cv. So Sweet 6*	2009[a]	JX267280
SOS8-TRV5	USA, OH	*Hosta sp.cv. So Sweet*	2009[a]	JX267281
SOS8-Tobra5	USA, OH	*Hosta sp.*	2009[a]	JX267285
SOS8-TRV1	USA, OH	*Hosta sp.*	2009[a]	JX267286
SOS8-TRV2	USA, OH	*Hosta sp.*	2009[a]	JX267287
SOS8-TRV3	USA, OH	*Hosta sp.cv. So Sweet*	2009[a]	JX267288
SOS8-TRV4	USA, OH	*Hosta sp.cv. So Sweet*	2009[a]	JX267289
SYM	UK	*Spinacia oleracea*	1985	D00155
Toya80	Japan	*Paeonia lactiflora*	2018[a]	LC553845
YZ	China	*Paeonia suffruticosa*	2016	KX345120
PEBV (SP5)	England	*Pisum sativum*	1964	X14006

* Shortened ID as it appears in Fig. 2 and 3

[a]Year of sample collection

[b]Vector

[c]Initially isolated from *P. pachydermus* from Scotland (Ploeg et al., 1992) and then cloned and used to infect other plant species.

Table S2. Sequences of CNSV isolates and Artichoke Italian latent virus used for phylogenetic analysis (ordered as they appear on the tree on Fig. 3). Sequences generated in this study are in bold font.

Isolate name	Country, state	Host Species	Year of submission to GenBank or isolation	GenBank accession number
OR Itoh 2	USA, Oregon	*Paeonia lactiflora Itoh*	2016[a]	MW328869
OR 117	USA, Oregon	*Paeonia lactiflora*	2016[a]	MW328863
Anhui	China	*Paeonia suffruticosa*	2021[a]	MZ576550
BJ	China	*peony*	2017[a]	MN253490
**MI-Plac-Gis1** (Gis1-2016)*	**USA, MI**	***Paeonia lactiflora***	**2016[a]**	**MK493790; MK521446**
OR Itoh 1 `	USA, Oregon	*Paeonia lactiflora Itoh*	2016[a]	MW328867
OR Itoh 3	USA, Oregon	*Paeonia lactiflora Itoh*	2016[a]	MW328871
AK 323	USA, Alaska	*Paeonia lactiflora*	2016[a]	MW328841
NY 51	USA, New York	*Paeonia lactiflora*	2016[a]	MW328859
AK 81	USA, Alaska	*Paeonia lactiflora*	2016[a]	MW328845
PO	South Korea	*Paeonia officinalis*	2020	LC577868
AR AHF	USA, Arkansas	*Paeonia lactiflora*	2019[a]	MW328847
AK 16	USA, Alaska	*Paeonia lactiflora*	2016[a]	MW328839
**MI-Plac-Gis2** (Gis2-2016*)	**USA, MI**	***Paeonia lactiflora***	**2016[a]**	**MK493791; MK521447**
NY 41	USA, New York	*Paeonia lactiflora*	2016[a]	MW328857
NY 6	USA, New York	*Paeonia lactiflora*	2016[a]	MW328861
OR 122	USA, Oregon	*Paeonia lactiflora*	2016[a]	MW328865
**MI-Plac-J1** (J1-2016*)	**USA, MI**	***Paeonia lactiflora***	**2016[a]**	**MK493795; MK521451**
**MI-Plac-G1** (G1-2016*)	**USA, MI**	***Paeonia lactiflora***	**2016[a]**	**MK493794; MK521450**
**MI-Plac-DP** (DP1-2016*)	**USA, MI**	***Paeonia lactiflora***	**2015[a]**	**MK493788; MK521443**
AD	South Korea	*Paeonia suffruticosa*	2014	MK512741
AK 50	USA, Alaska	*Paeonia lactiflora*	2016[a]	MW328843
Won, Paeonia nepovirus A	China	*Paeonia lactiflora*	2015[a]	MH898603
AR MW	USA, Arkansas	*Paeonia lactiflora*	2016[a]	MW328853
**MI-Plac-I1** (I1-2016*)	**USA, MI**	***Paeonia lactiflora***	**2016[a]**	**MK493796; MK521452**
**MI-Plac-Y2** (Y2-2016*)	**USA, MI**	***Paeonia lactiflora***	**2016[a]**	**MK493793; MK521449**
**MI-Plac-Y1** (Y2-2016*)	**USA, MI**	***Paeonia lactiflora***	**2016[a]**	**MK493792; MK521448**
Ho40	Japan	*Paeonia lactiflora*	2020	LC553843
**MI-Plac-Gis2** (Gis2-2015*)	**USA, MI**	***Paeonia lactiflora***	**2015[a]**	**MK493789; MK521444**

NY 33	USA, New York	*Paeonia lactiflora*	2016[a]	MW328855
AR K	USA, Arkansas	*Paeonia lactiflora*	2019[a]	MW328851
AR CX	USA, Arkansas	*Paeonia lactiflora*	2019[a]	MW328849
pt077-sec-6	China	*Solanum lycopersicon*	2019	MN814315
CO	South Korea	*Cnidium officinale*	2020	MN905738
Lily-1	Australia	*Lilium longiflorum*	2010	JN127336
DJ	South Korea	*Daphne odora*	2016	MK521837
CNSV-A	China	*Medicago sativa*	2019	BK010916
CNSV	New Zealand	*Paeonia lactiflora*	2008	EU741695
CNSV	Japan	*Cycas revoluta*	2001	AB073147
Artichoke Italian latent virus, AILV-V	Bulgaria	*Vitis*	2016	NC_043684

* Shortened ID as it appears in Fig. 2 and Fig. 3
[a]Year of sample collection

File S1. Recommendations for the management of the UMNA peony collection and other *Paeonia* germplasm banks and public gardens.

Together with our collaborators, we have documented the existence of additional viruses in the UMNA peony collection [42,20–22]. Given that the UMNA *Paeonia* collection has an irreplaceable horticultural and scientific value, continuous screening and eradication of potentially pathogenic viruses for the development and maintenance of a virus-free collection is of upmost importance. The fact that CNSV is often asymptomatic, or symptoms are not clearly indicative of infection, challenges the diagnosis and control of this virus. Furthermore, the common methods used on agricultural crops to eradicate viruses, such as the destruction of plants and the replacement from stocks, is not concordant with the goal of maintaining the historic and often unreplicated diversity of peony cultivars in this reference collection, so other measures should be considered [2,12], which we summarize here. Among the challenges of controlling these viruses at the UMNA peony living collection is the inherent need to add and share specimens with other similar collections. The UMNA peony collection is both a germplasm collection of historical peony cultivars, and a display public garden. Most of the cultivated peonies are vegetatively propagated (through vegetative root parts) and specimens acquired from or shared with other institutions could carry viruses, especially in plants

that are asymptomatic (particularly for CNSV). Another challenge is the lack of knowledge regarding the natural vectors of these viruses in the environment. For TRV it is known that nematodes of the genera *Trichodorus* and *Paratrichodorus*, which have a worldwide distribution, may be vectors of this virus [31]. However, although most of nepoviruses are known to be primarily transmitted by nematodes [31], we do not know for certain that CNSV is transmitted by a nematode or the taxonomic identification of potential nematode vectors. Although it is common practice to ensure that nematodes are not transported with roots used for propagation, knowing the vectors transmitting these viruses at UMNA would allow to design effective measures to control their spread within the collection. Possibilities of CNSV transmission via pollen and mites have been suggested and should be further investigated [54]. Finally, the control of these viruses in the UMNA peony collection would need to be conducted following strict regulations to prevent the loss of biodiversity in the collection. The results of our work highlight the threats of TRV and CNSV infections not only to germplasm *Paeonia* collections, but also to the economically important industry of cultivated peonies. To develop and maintain a TRV and CNSV-free peony living collection that allows long-term preservation of the genetic diversity of cultivars, we propose the following steps at UMNA (and other gardens in the peony conservation network): 1) assess viral infection throughout the collection; 2) decontaminate plants to develop a virus-free stock, beginning with the rarer cultivars, 3) create an *in vitro* repository collection of laboratory propagated virus-free peonies, and; 4) address the feasibility of establishing a virus-free field collection if the current site cannot be decontaminated or these and other emergent viruses inactivated.

1. To assess the extent of TRV and CNSV viral infections in the UMNA peony collection, it is necessary to perform molecular analyses of every accession plant in the collection - symptomatic or asymptomatic. Nematode vectors need to be identified and their abundance quantified by soil samples for each planting bed. To determine the extent of infection of the two viruses and quantify cultivar susceptibility all specimens in the UMNA collection should be tested. This will be an important base-line data for monitoring and breeding programs.

2. The UMNA peony living collection (and the few other major peony collections), includes historic cultivars,

most of which cannot be found anywhere else in the world or are extremely rare. Therefore, a viral infection of particular plants could represent an extinction threat for these lineages. Thermotherapy can be used as a first step in decontaminating rare peonies. To avoid further viral infections, we also recommend testing all incoming plants and those shared with other institutions using molecular methods.

3. The long-term conservation of these cultivars may be best achieved by establishing a repository of laboratory propagated cultivars that can serve as a reservoir of the virus-free germplasm. Cryopreservation methods to maintain *in vitro* cultures (micropropagation) have been successfully used in other plant cultures [61-64], including historical varieties [65]. However, the challenge for successful micropropagation of historic herbaceous peonies is their recalcitrance to *in vitro* methods. While some researchers have attempted some micropropagation protocols for *P. lactiflora* cultivars [66–69], protocols must be optimized for different cultivars [70], due to the biology and secondary metabolites content differences between them. Verevchik (2017) [71] further explored methods for successful *in vitro* culture in this recalcitrant group. A multi-institutional collaboration, for example within with the National Clean Plant Network, will be beneficial.

4. The need for virus-free living collections requires long-term assessment of plant accessions. Decontaminating field sites of living collections that function as public gardens is challenging because they may be vulnerable to re-infection due to continued public access [72]. It is not feasible to simply eliminate infected plants from the collection, as they may represent the last known plants of a particular cultivar and their removal significantly reduces the conservation value of the collection and its mission. To guide decision-making of a management strategy it would be important to develop a rubric to explicitly consider losing unrecoverable diversity against the risk of maintaining rare or unique cultivars that are infected with viruses.

References for File S1 are included in the Reference list in the main text.
